# Characterizing governance models for upscaling wetland restoration

**DOI:** 10.1007/s00267-025-02132-2

**Published:** 2025-03-03

**Authors:** Arnaud Terrisse, Michael Karner, Julia Kaufmann, Lisa Ernoul

**Affiliations:** 1 Plan Bleu (Regional Activity Centre - UNEP/Mediterranean Action Plan) - Tour la Marseillaise - 2 bis, Boulevard Euroméditerranée Quai d’Arenc, 13002 Marseille, France; 2 Tour du Valat, Research Institute for the Conservation of Mediterranean Wetlands, Le Sambuc, 13 200 Arles, France

**Keywords:** Governance models, Ecological restoration, Wetlands, Upscaling

## Abstract

Governance is a key element for effective conservation, sustainable management and restoration of ecosystems. Improving governance is essential for upscaling restoration actions around the world. Governance systems were studied in eleven on-going European wetland restoration sites using a two-step process. First, an in-depth examination of seven sites from six countries was made using key informant interviews. This information provided the basis for developing four governance models (Monocentric, Polycentric, Community-based and Networking). Most of the sites presented a dominant governance model, but also incorporated some dimensions of the other models to a lesser degree. The models were tested and evaluated in an additional seven sites in five countries. The analysis revealed that the governance models were highly subject to the geographical features, land ownership structures and different histories of the sites. Strengths, weaknesses, and supporting and limiting factors were associated with each model. This research shows how governance models are adapted to site specific conditions and how these adaptations can be used to enhance governance processes in existing sites, upscale restoration efforts or set the groundwork in new restoration sites.

## Introduction

Wetlands are among the most dynamic ecosystems on the planet. Over 40% of the world’s species live in freshwater wetland habitats, which are essential not only for the species living in them, but also play a vital role for migratory species (Perennou et al. 2020). They maintain ecological processes, providing key ecosystem services, including water storage and aquifer recharge, water quality regulation and the assimilation of pollutants and excess nutrients, the deposition of excess sediment and the capture and long-term storage of carbon (Mitsch et al. 2015). Salt marshes, healthy mires, peatlands and riparian zones constitute the main European wetland habitats in terms of carbon storage. It has been estimated that over 87% of inland wetlands were lost between 1700 and 2000. Between 1970 and 2008, losses accelerated in coastal areas, with a decline of over 30% (WWF 2019).

The main driver of European wetland degradation and loss since 1970 has been land use change, including urbanization and transformation of wetlands into agricultural lands (Metzger et al. 2006). These changes have had a profound impact on wetland biodiversity. Wetland dependent species have declined more than those in other biomes, and an increasing number are facing extinction (Convention on Wetlands 2021). The Black Sea and Mediterranean regions have experienced the highest rates of relative wetland loss in Europe (Maes et al. 2020). Additionally, decreasing precipitation and increasing temperatures predicted by current global climate change scenarios (Barredo et al. 2016) could lead to further losses and conservation challenges. Coastal wetlands (suffering from sea-level rise and coastal erosion) as well as Arctic and mountain wetlands (due to the shrinking cryosphere) are especially at risk due to climate change (Convention on Wetlands 2021). Wetland degradation is exacerbated by a lack of a comprehensive policy framework, inadequate definitions and representation of wetlands in different classification systems, and the lack of proper impact assessments prior to land use changes (IPBES 2018).

Wetland restoration is promoted as a tool to counteract these negative trends (Cliquet et al. 2022). The restoration of wetlands is seen as a possible solution to provide essential ecosystem services for biodiversity and humankind, but must incorporate public policy, governance schemes, and corporate responsibility in order to have the desired impacts (IPBES 2018). Despite the best intentions, wetland restoration is open to wide interpretations with differing theoretical issues which can result in diverging visions and eventual conflicts (Aggestam 2013). In order to reduce these obstacles, it is important to create and adapt governance frameworks that are appropriate in each specific context.

Environmental governance englobes the rules, laws, policies, structures, networks and procedures that create the dynamics and behaviors of stakeholders in the use and management of natural resources (Bennett and Satterfield 2018). Appropriate governance is essential for the conservation, sustainable management and restoration of natural resources. Governance has been shown to be a determining factor in the effectiveness of conservation efforts and the enhancement of benefits to human well-being (Springer et al. 2021). Successful resource management requires both science-based ecological techniques and a cooperative political and socio-cultural environment (Gumiero et al. 2013). Important benefits can be derived when ecological restoration is developed and implemented through the involvement and interaction of different stakeholders, working at different levels and scales and incorporating cross sectoral interests (Richardson and Lefroy 2016). Socio-economic and government systems have been identified as the main barriers to successful ecological restoration in Europe because they can cause conflicting interests between stakeholders. Low political priority given to environmental issues and insufficient funding also hinder restoration action (Cortina‐Segarra et al. 2021). The use of governance tools such as site-specific and voluntary environmental contracts for wetland can improve the overall effectiveness of wetland restoration (Ernoul et al. 2021).

The European Union (EU) Green Deal comprises a package of policy initiatives, including the EU Biodiversity Strategy for 2030 and the recently approved Nature Restoration Law, that aim to set the path for the EU to green transition, with the final goal of reaching climate neutrality by 2050 and tackling biodiversity loss for increased prosperity, sustainability and resilience. Part of this transition involves effectively upscaling nature restoration, including wetland restoration. Upscaling takes into account both increasing the intensity and surface area of restored sites. Part of upscaling involves testing and adapting governance models across the EU. This can provide lessons learnt for other areas dealing with human-nature interactions, where informed discussions between and among local communities, stakeholders and decision-makers are required to take action on management planning for biodiversity, carbon stocks/balances, ecosystem services and resources (Albert et al. 2021). Improved policies and integration of best practices based on lessons learnt from wetland restoration are necessary to identify innovative policy and governance pathways that support large-scale wetland restoration (Sánchez-Arcilla et al. 2022).

Wetland governance can take many different forms and occur at multiple levels: horizontally (between government agencies or economic sectors) and vertically (through institutional hierarchy). This multilevel governance can operate formally (through laws or legal contracts and agreements) or informally (based on relationships and trust) (Borrini-Feyerabend et al. 2013). In the context of the EU Green Deal, the EU funded the WaterLANDS (Water-based Solutions for Carbon Storage, People and Wilderness) project aimed at upscaling the restoration of wetlands across Europe by co-creating a new paradigm of restoration best practices based on four key pillars: ecology, community, governance and finance (https://waterlands.eu/). The project involves 15 experienced wetland restoration sites that capitalize upon their experiences and 6 new restoration sites that provide the ground to test new practices. In the framework of the WaterLANDS project, this article analyzed and characterized different governance models used in wetland restoration from 12 European countries (Ireland, Spain, France, Italy, Bulgaria, Poland, Sweden, Germany, England, Estonia, The Netherlands, and Poland). The objective is to share the results and lessons learnt to inform decision-makers, local communities, and restoration practitioners on possible methods to improve governance in wetland restoration projects and to promote upscaling efforts.

## Theoretical governance framework

**Fig. 1:**
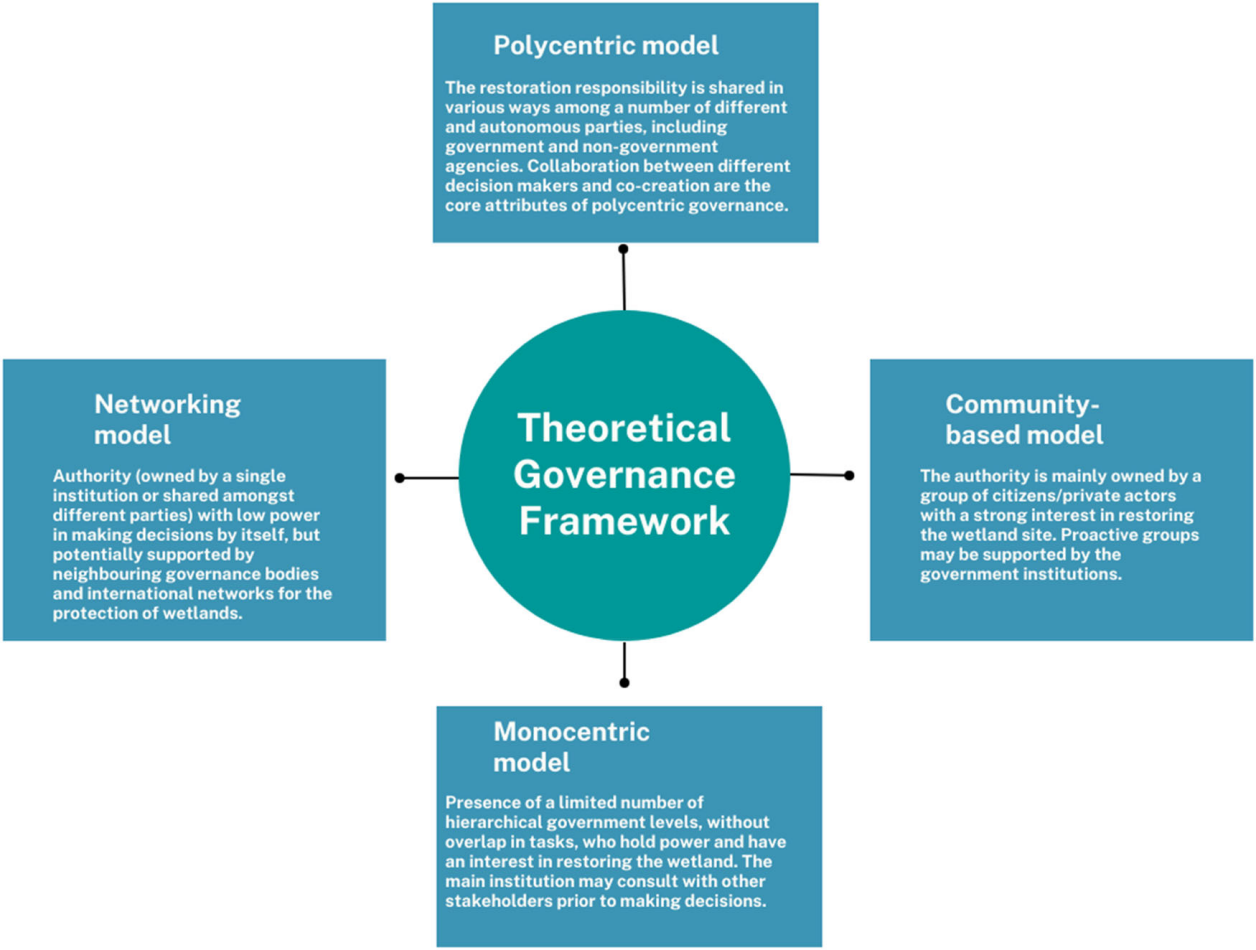
Four theoretical governance models identified among restoration sites in Europe

Four main models were identified, based on classifications by Carlisle and Gruby and Termeer et al. (2019; 2010) and the experiences of the 15 restoration sites involved in the WaterLANDS project: monocentric, polycentric, community-based and networking governance models (Fig. 1).

In the *Polycentric Governance Model*, decisions are made among multiple independent actors, including governmental and non-governmental organizations. The main features of polycentric governance are collaborative decision-making processes and co-creation mechanisms. The EU identified polycentric governance as a driver for successful Nature-based Solutions (NbS) implementation (EC 2003) and provided clear guidance encouraging collaborative planning through different policies, e.g., the Flood Directive, the Water Framework Directive and the Public Participation Directive. These systems generally operate at different scales and across sectors to better address the complexity of social-ecological systems. Supporting factors for this model include long-term behavioral changes concerning cooperation and trust built between stakeholders (Asley et al. 2008). But cooperation requires time and the commitment of all the stakeholders involved (Roulin et al. 2017). Most importantly, polycentric governance requires strong institutional arrangements and structural funding to pursue common goals, manage resources responsibly and conserve biodiversity effectively and legitimately (Andersson and Ostrom 2008; Young et al. 2016). Moreover, Thaworn et al. (2010) have underlined the importance of evaluating and strengthening existing arrangements to adapt to changes in social-ecological systems. Some lessons from polycentric governance and NbS have been identified. For instance, the Isar River restoration in Munich implemented from 2000 to 2011, involved institutions and local stakeholders in the co-design of NbS through an intensive and longstanding participatory process. The Isar-Plan governance decision-making process was led by different decision centers and the interaction of different sectors at different geographical scales. This governance model helped increase socio ecological resilience (Zingraff-Hamed et al. 2019).

The *Monocentric Governance Model* is a hierarchical governance system characterized by the dominance of a single actor in decision-making processes (often a governmental actor at the national level) (Van Zeijl‐Rozema et al. 2008). Most European countries operate a three-tier system with national, provincial and municipal levels, which determine the level where decisions are made and can also be characterized as multi level governance where dispersal and redistribution of powers and competences to different levels of policymaking activity take place (Paul Stephenson 2013). However, power is often concentrated at the top level, which controls and constrains the lower levels of government (Termeer et al. 2010). The central authority sets goals and implements policies in a top-down manner. Some authors have argued that public participation may be easier in monocentric systems than in polycentric systems, since it is easier to provide feedback to the public if there is only one (governmental) center of power than if there are many (Morrison et al. 2019).

The *Community-based Governance Model* focuses on community-based management systems. A community-based approach can support livelihoods and biodiversity while reinforcing the meaningful participation of local indigenous communities, but also their values, cultures and institutions (Esmail et al. 2023; Parks and Tsioumani 2023). Authority is given to a group of deeply invested citizens or private actors. The groups may be supported by formal institutions such as local authorities or government agencies. In many community-based models, citizens lead governance efforts, creating a vision for the area, and collectively managing projects (Scarlett and McKinney 2016; Edelenbos et al. 2021). This model is often found in small-scale projects with locally focused issues and has been shown to motivate higher levels of participation and engagement from local stakeholders (Marshall 2009).

The *Networking Governance Model* uses support from various governance bodies to enhance decision-making power. Network governance can take form when stakeholders realize that the problems cannot be resolved without outside support (Scarlett and McKinney 2016). This model complements, rather than replaces, polycentric or monocentric governance models. Network governance enables multiple forms of environmental and organizational leadership (Imperial et al. 2016). Successful network governance can enhance environmental management efforts by promoting inclusive and equitable partnerships. The benefits of network governance, which include flexibility and inclusive participation, also present distinct challenges, such as network capture, shifting stakeholder boundaries and competing knowledge claims (Bixler et al. 2016). This resonates with adaptive governance systems which often self-organize as social networks with teams and actor groups that draw on various knowledge systems and experiences for the development of a common understanding and policies (Folke et al. 2005).

## Materials and methods

This research was conducted in three steps: a) survey questionnaires for key informants from ongoing wetland restoration projects across Europe, b) in-depth interviews in selected restoration sites and c) secondary questionnaires to validate and triangulate governance model schemes.

### Survey questionnaires and selection of in-depth study sites

**Fig. 2:**
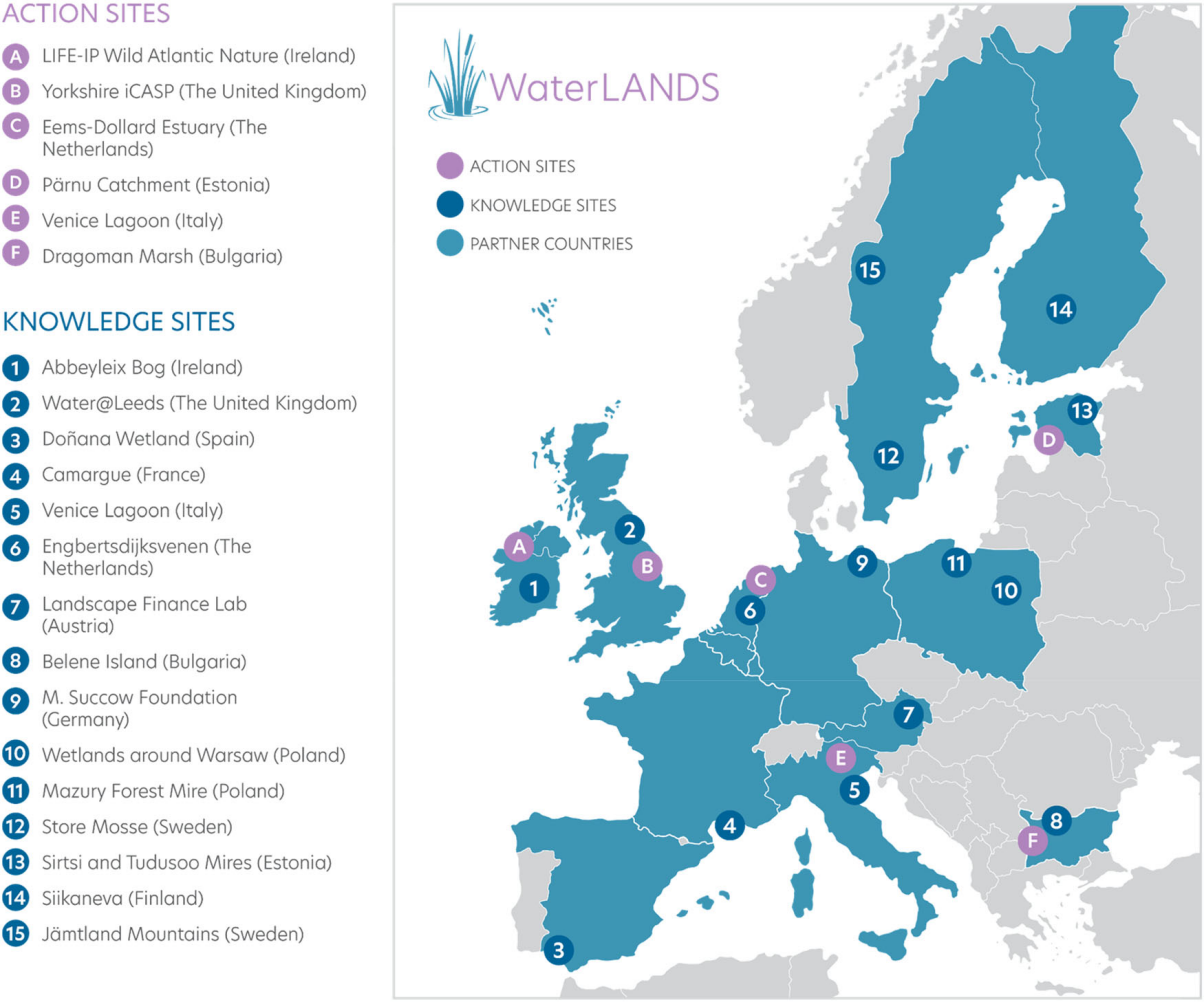
Map of knowledge and action sites involved in the WaterLANDS project

The WaterLANDS project incorporates 15 sites around Europe that have been identified as “Knowledge Sites” (Fig. 2). These sites have undergone previous wetland restoration actions over the last decade and their experiences can thus serve as examples for lessons learnt and upscaling restoration activities. This research project sent a first questionnaire to representatives of the 14 “Knowledge Sites” in May 2022. The 14 sites are used to gain experience and lessons learnt from past restoration projects and are at the heart of the knowledge sharing encompassed in the WaterLANDS project. This research began studying seven sites that exhibited complex governance settings for which an in-depth analysis would provide insight to create actionable recommendations tailored to diverse restoration contexts. The other seven sites were added to test the models identified and to help get crucial information for the development of a theoretical governance framework. It is important to note that the Finish site Siikaneva (shown below in the map) has not been included in this study because this site was not restored and does not provide any governance lessons. It is a pristine wetland site used as a reference as part of the European Carbon Monitoring Network (Integrated Carbon Observation System - ICOS).

This first questionnaire aimed to select sites with the best representative diversity in terms of governance (a copy of the questionnaire can be found in the supplementary material). The site selection was based on a preliminary analysis of the existing governance structures (including governance systems, and number and diversity of stakeholders), types of wetlands and geographical scope. A total of 7 sites from 6 countries were selected (Table 1).

**Table 1:** Restoration sites that participated in the research and involved in the WaterLANDS project

	Site name	Site status	Coordinating institution and type of stakeholder	Country
Selected sites for in-depth analysis	Abbeyleix Bog	No status	Irish Rural Link - NGO	Ireland
	Doñana wetlands	National Park	Consejo Superior de Investigaciones Científicas (Spanish National Research Council) - Research & Academia	Spain
	Camargue	Camargue Regional Natural Park	Tour du Valat - Research & Academia and land manager	France
	Venice Lagoon	Special Protection Area (IT3250046) and Special Conservation Zones “Northern Lagoon of Venice” IT3250031 and “Central-Southern Lagoon of Venice” IT3250030 under the Birds and Habitat Directives. The “City of Venice and its lagoon” is a UNESCO World Heritage site	We are Here Venice - NGO	Italy
	Belene Island	Ramsar site, Persina Nature Park, Natura 2000 site	WWF Bulgaria - NGO	Bulgaria
	Wetlands around Warsaw (Całowanie Fen and Kampinos)	Kampinos wetlands are part of the Kampinos National Park (KNP) territory. Całowanie Fen is part of the Masovian Landscape Park (MLP)	CMoK, Kampinoski National Park - Research & Academia/Public Authority	Poland
Additional sites validating the models	Jämtland Mountain	Vålådalen Nature Reserve, Natura 2000 site	University of Uppsala - Research & Academia	Sweden
	Store Mosse	National Park, Ramsar site	University of Uppsala - Research & Academia	Sweden
	Karrendorf Meadows (Greifswald)	No status	Michael Succow Foundation - Private Foundation	Germany
	iCASP Yorkshire	The Great North Bog includes four National Parks, three National Landscapes and the proposed South Pennines Park	University of Leeds - Research & Academia	England
	Tudu-Sirtsi	Sirtsi Wetlands Nature Reserve	Estonian Fund for Nature, University of Tartu - NGO/Research & Academia	Estonia
	Engbertsdijksvenen	National Ecological Network, Natura 2000 site	Staatsbosbeheer - Public Authority	The Netherlands
	Mazury Forest	Mazury Landscape Park, Natura 2000 site	University of Warsaw - Research & Academia	Poland

### In-depth interviews and creation of governance models

In November 2022, in-depth interviews were conducted virtually with representatives from the seven selected sites: Abbeyleix Bog, Persina Nature Park - Belene Island, Camargue former saltworks, Doñana wetlands, Kampinos wetlands/Bagno Calowanie and the Venice Lagoon. The sites selected covered both inland and coastal wetlands, and represented three biogeographic regions (Continental, Mediterranean, Atlantic) and six countries. The interviews were adapted to each site based on their responses from the first questionnaire (Goeldner-Gianella and Humain-Lamoure 2010). Each interview lasted approximately 1.5 h and involved one to three key informants from each site. The interviews were recorded for later analysis.

### Second questionnaire and validation of governance models

An additional synthetic questionnaire was prepared for seven other “Knowledge Sites” from five other countries. The second questionnaire was sent to key informants from each site (*n* = 14) and aimed to obtain specific information for the development of a Theoretical Governance Framework. In the first section, recipients were asked open-ended questions on the governance scheme in place at their wetland. They then responded to questions about their governance model, types of support obtained from global, European and national policies, and the availability of funding. Finally, the recipients were requested to assess the supporting and limiting factors of governance resulting from the cross-analysis of the original seven sites. This assessment was made using a semi-quantitative scoring system (0 - no relevance/low relevance; 1 - medium relevance; 2 - high relevance). The survey analysis triangulated qualitative data analysis and standard descriptive statistics including counts, percentages and frequency (Dey 1993; Kitchin and Tate 2000).

Lastly, each governance model was further analyzed using a SWOT analysis (identifying the strengths, weaknesses, opportunities and threats) (Puyt et al. 2020) and the supporting and limiting factors were roughly associated with the most predominant governance model (see supplementary material for the SWOT analysis).

## Results

### Governance Framework to guide wetland restoration upscaling

**Fig. 3:**
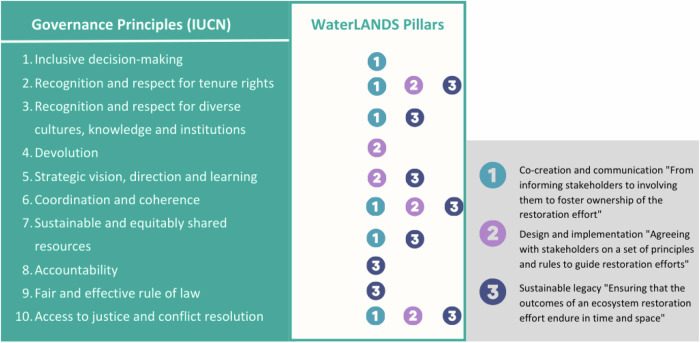
WaterLANDS pillars and their relationship with the IUCN’s governance principles

Three pillars were developed within a Theoretical Governance Framework based existing conceptual frameworks, including International Union for Conservation of Nature’s (IUCN) governance principles (https://www.icgn.org/sites/default/files/2021-11/ICGN%20Global%20Governance%20Principles%202021.pdf), to gather elements that contribute to the effective, fit-for-purpose governance of wetland restoration efforts. The ten principles in the IUCN framework provide a substantial theoretical contribution and compose the Natural Resources Governance Framework (NRGF), a knowledge product created as a tool to improve governance for equitable and effective conservation. The NRGF can be applied to wetlands since its criteria have been intentionally kept general for use in different contexts and for different types of natural resources and include aspects of transformative governance for successful wetland restoration and the notions of co-creation and sustainable legacy, which all contribute to the upscaling of restoration (Fig. 3). Transformative governance can be described as the set of formal and informal (public and private) rules, rulemaking systems and actor networks at all levels of human society that enable transformative change (Visseren-Hamakers et al. 2021).

### First questionnaire: commonalities and differences between the sites

The first questionnaire revealed diverse heterogeneous models of governance, mainly due to different geographical features of the sites (e.g. wetland surface area), ownership type (private land, state land) and historical context (land use change and anthropogenic pressures, previous management experiences). Restoration actions in the sites were initiated by a variety of different factors. One common initiator for several sites was a change in land ownership and land-use change (from commercial exploitation to conservation). This often marked a decisive transformation in governance models. In Camargue, for example, the acquisition of the site by the Conservatoire du Littoral and the end of salt production were key factors in launching a large-scale restoration program. Similarly, at Abbeyleix Bog, a community-led company (Abbeyleix Bog Project Ltd) began a restoration program after negotiating a 50-year lease with the Bord Na Mona semi-state company that used the site for fossil peat extraction. Additionally, risks associated with climate change were cited in many sites as increasingly relevant for orienting and sparking restoration actions. In Camargue, the ecological restoration program (hydrological reconnection of water bodies) strengthened ecosystem functioning to improve the resilience to climate change. In the Venice Lagoon, protecting the city and other minor islands from sea level rise supported restoration activities preserving the functioning of the lagoon’s ecosystem and pursuing the environmental objectives set by several European Directives including the *Water Framework Directive* (2000/60/) and the *Habitats Directive* (92/43/EEC). In Belene, the international dimension of restoration was a key feature of this wetland restoration activity. An overarching vision for the entire lower Danube River stretch was set up before the restoration started, through the international agreement for the lower Danube Green Corridor. This agreement sets up ambitious objectives for the whole area that the Global Environmental Fund contributed to achieve locally in the Belene Wetland. The involvement of international experts from the World Bank was also decisive since they brought their knowledge and contributed to an effective design of restoration measures.

Conversely, fragmented ownership of wetlands and limited possibilities for funding land purchases were highlighted as common barriers for restoration (Calowanie and Doñana wetlands). Most of the sites evoked the poor recognition of the ecological value of wetlands, with severe losses occurring in the last century. The construction of dykes (creating artificial hydrological conditions) and drains were common practices used for peat extraction (Abbeyleix Bog) or agricultural production (Belene, Calowanie, Camargue). As a result, the progressive drying of wetlands and decreasing water tables were observed in many of the sites. Periods of political instability were finally mentioned as a threat (Belene), relegating environmental protection to the lowest priority for governments.

The first questionnaire identified factors that contributed to support (Table 2) or hinder (Table 3) ecological restoration in each site.

**Table 2:** Governance-related factors that support ecological restoration

Supporting factors	Abbeyleix Bog	Doñana	Camargue	Venice Lagoon	Belene Island	Kaminos	Całowanie
A multi stakeholder committee (decision-makers and other interested parties)	x	x	x		x		
Proactivity of citizens’ associations	x						x
Land acquisition by authorities			x				x
Governmental support (political, financial)	x			x			
Recognition of Nature-based Solutions and adaptive management			x				
Research institute involvement		x					
Recognition of intangible values (cultural and aesthetic)	x						
Establishment of visitor centers, hiking and biking trails	x						
Early citizen involvement	x						
Participation in EU funded projects (especially LIFE)		x	x	x		x	x

**Table 3:** Governance-related factors that hinder ecological restoration

Limiting factors	Abbeyleix Bog	Doñana wetlands	Camargue	Venice Lagoon	Belene Island	Kaminos	Całowanie
Lack of an overarching strategy and long-term vision	x	x		x			
Lack of a comprehensive approach to managing the site at a broad spatial scale							x
Lack of dialog with national agencies managing different sectors							x
International political instability					x		
Multiple governance bodies with inefficiencies				x			
Conflicting interests and land uses		x	x	x			
Scattered land ownership with varying tenure rights and limited interest		x					x
Change in governance and political vision over time		x		x			
Skepticism due to lack of understanding		x	x		x		x
Late/ poor involvement of stakeholders		x		x			
Lack of funding sources					x	x	x

Multi-stakeholder committees were identified as a supporting factor for effective restoration. This demonstrated the importance of coordinated action taking into account multiple perspectives. The Council for Doñana Wetlands is one example of this type of committee. Its international recognition allows it to address the threats posed by the overexploitation of aquifers. Participation in European funded initiatives, especially LIFE projects, which is the main EU funding instrument for the environment and climate action, was also considered an important supporting factor with the promotion of participatory approaches, communication and awareness raising. LIFE projects had been implemented in the two Polish sites, Camargue and Venice Lagoon.

A recurring limiting factor in many of the sites was the lack of an overarching strategy and long-term vision (Abbeyleix Bog, Doñana wetlands and Venice lagoon). Conflicting interests and land use preferences from different actors were also frequently mentioned (Doñana wetlands, Camargue and Venice lagoon). In addition, the skepticism and lack of understanding of the benefits for restoration were also seen as limiting factors. This can be seen in the Camargue and Belene Island, where concerns about dike removal to support restoration was put into question.

### In-depth interviews and development of governance models

An in-depth analysis of the first seven sites using key informant interviews provided the basis for classifying the heterogeneous governance systems into broader categories.

### Second questionnaire: governance-related supporting and limiting factors

**Fig. 4:**
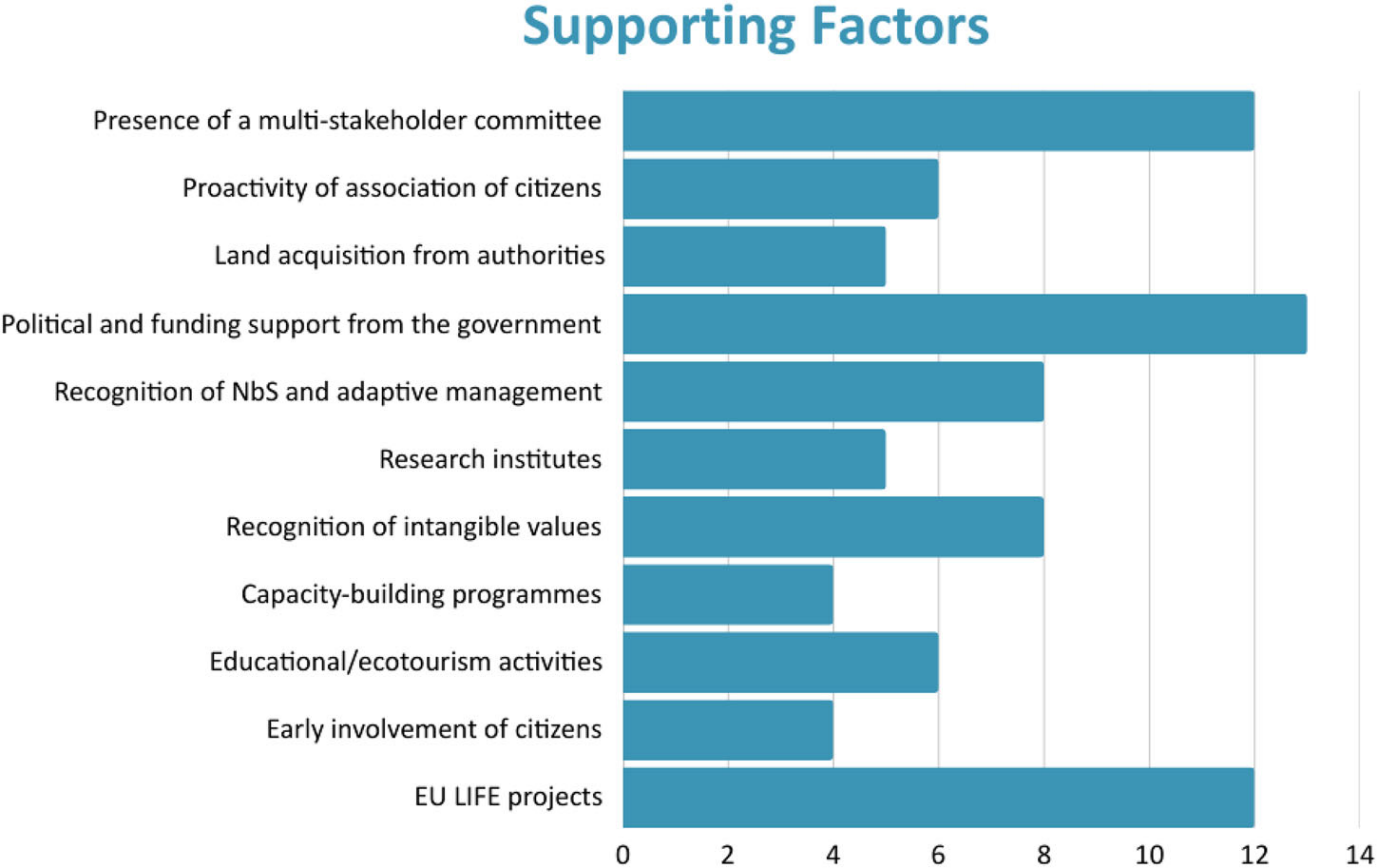
Supporting factors for ecological restoration ranked according to importance

**Fig. 5:**
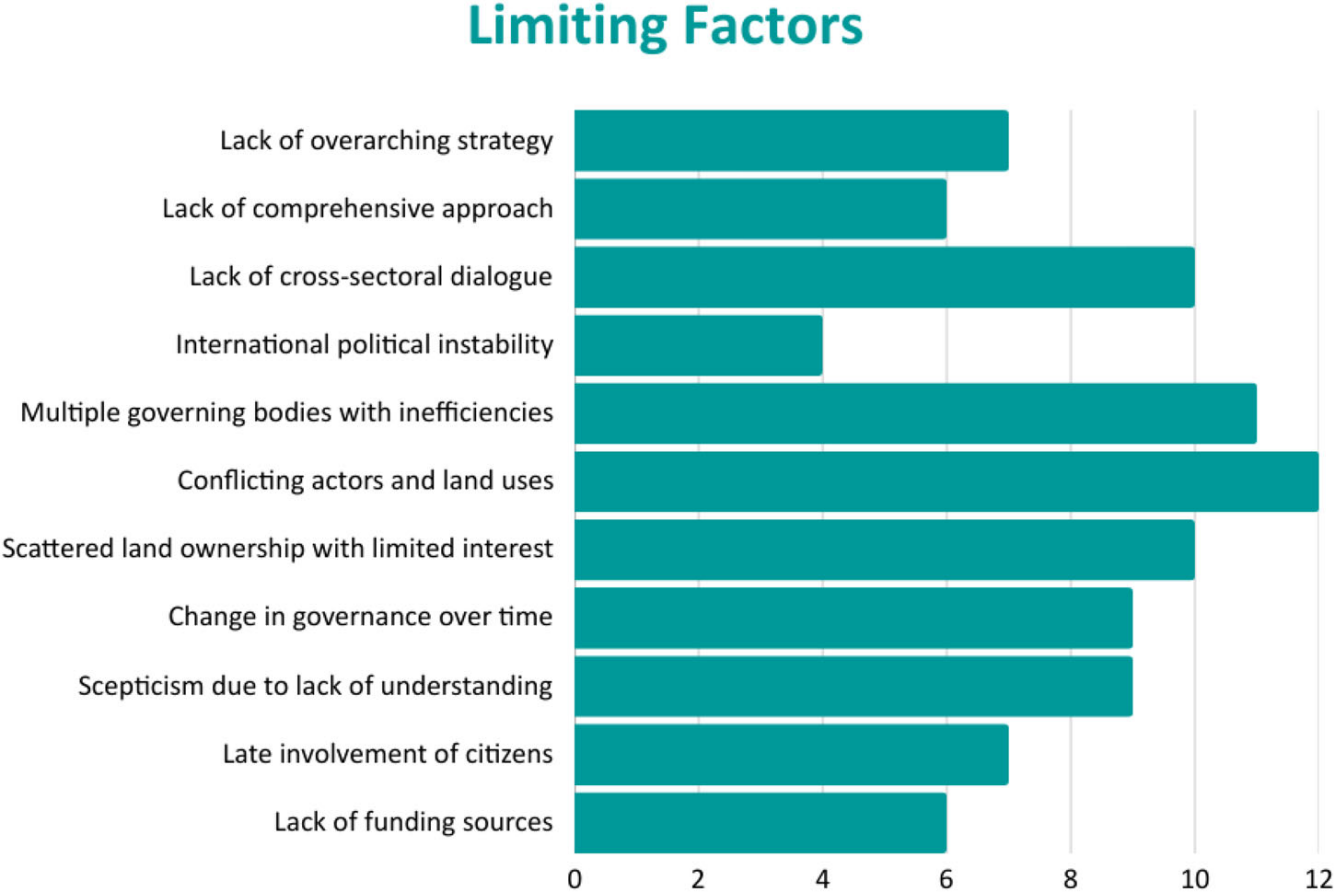
Limiting factors for ecological restoration ranked according to importance

Each of the sites evaluated the importance of the supporting (Fig. 4) and limiting (Fig. 5) governance factors according to their respective importance (scores: 0 = no/ low relevance; 1 = medium relevance; 2 = high relevance).

The assessment was conducted using a semi quantitative score system. The total scores were calculated from the case studies as the sum of individual scores. The three governance-related supporting factors that ranked the highest overall scores were political and financial support from the government (13), presence of multi-stakeholder committees (12), and involvement in European funded projects (12) (Fig. 3). The highest ranked limiting factors were conflicting interests and land use preferences of different stakeholders (12), coordination between multiple overlapping governance bodies (11), and scattered land ownership and a lack of cross-sectoral dialog (10) (Fig. 4).

## Discussion

The restoration sites were categorized into four theoretical models, but governance structures vary and often combine different characteristics of multiple models. The classification into specific models is useful to analyze the governance systems and to make recommendations, but it is important to note that the classifications are often fuzzy with characteristics from two or more models. Although polycentric and monocentric systems represent the ends of the governance spectrum, they can coexist in complex, intertwined ways (Morrison et al. 2019). We have seen in this research that community-based and networking models are not substitutes for the latter, and can also develop within existing governance contexts. They should therefore not be considered as entirely separate from polycentric or monocentric systems.

Most of the restoration sites were broadly classified as polycentric, with wetlands managed by multiple independent governmental and non-governmental actors who collectively shape the decision-making process. One example of polycentric governance is in the Camargue, where the restoration processes are coordinated by three co-managers (private and public actors), in collaboration with a state agency (who is also the landowner). Polycentric governance often consists of multi-stakeholder committees involving a multitude of stakeholders, such as in the case of Doñana. This polycentric model demonstrates how regional authorities can work in close collaboration with a local council made up of 32 stakeholders *(Patronato de Donaña)* including local landowners, farmers and conservationists. The restoration activities are collectively managed at different administrative levels, and include the participation of different organizations, social and economic actors, and the scientific community. Although the Venice Lagoon presents some elements of the polycentric model with responsibilities shared at multiple levels (national, regional and local), the system suffers from an uneven distribution of power among key actors and limited cooperation, with most funds managed at the national level and a single concessionaire carrying out public works. These limits in cooperation and stakeholder participation have created a system characterized as hierarchical and monocentric (Munaretto and Huitema 2012).

The Abbeyleix bog restoration project is a typical example of a community-based model. It is a community-led non-profit organization, driving decision-making processes. The initiative evolved from a local residents’ association dedicated to preserving and restoring the bog. Today the site is managed and continues to be restored with a Technical Advisory Group composed of landowners, representatives of national and county institutions, an independent conservation organization, and community-appointed members. Further East, Belene Island is a good example of the networking model. Located on the border between Romania and Bulgaria, the restoration effort was guided by an international agreement signed in April 2013, setting ambitious goals for the region. This was complemented by international organizations (Global Environment Facility and World Bank) providing expertise and funding to facilitate restoration efforts.

The SWOT analysis (identifying the strengths, weaknesses, opportunities and threats) (Puyt et al. 2020) and the associated supporting and limiting factors for each governance model can inform the design and scaling-up of restoration activities. Here, we concentrate on the strengths and weaknesses of each model and the associated factors (supporting and limiting) that were identified among the sites. The main strengths identified in polycentric governance systems are their potential “adaptive capacity” to adjust to socioeconomic and ecological change (Carlisle and Gruby 2019). This adaptability is essential for restoration activities as it allows for the activities, roles and responsibilities to be continuously updated based on the evolving conditions at the site (adaptive management). Another advantage is “institutional fit,” where governance structures are tailored to the specific needs of the area (Carlisle and Gruby 2019), allowing for better management of complex ecological systems (often through the presence of a multi stakeholder committee). Multiscale governance allows local decision-makers (governmental and non-governmental) to respond to change quickly, while providing support from higher decision-making levels to respond to broader challenges. The cross-sectoral cooperation found in polycentric governance enables integrated management, combining knowledge from different stakeholders (including research institutes and local citizens). In addition, these systems can reduce the risk of institutional failure, since different partners can complement each other (Munaretto and Huitema 2012). Polycentric models may take time to find agreement on a common vision, goals, and methods for wetland restoration. However, these preparatory phases could allow for processes to advance faster and more smoothly during the implementation phase.

Some weaknesses identified in the polycentric governance system are associated with joint decision-making. When decision-making is shared among different governmental and non-governmental actors, there could be a diffusion of authority and responsibility, underscored by potential problems in terms of accountability. Effective polycentric governance requires strong collaboration across different levels of governance and a strong culture of cooperation and trust (Munaretto and Huitema 2012). Coordinating multiple institutions is challenging, and poorly designed collaboration mechanisms reduce the effectiveness of polycentric governance. This can cause dysfunctional cooperation, costly transactions, and conflicts between actors (Mudliar 2021). Polycentric governance launched through projects can also lack funding over time, leading to a less comprehensive approach and limitations in developing overarching strategies.

Monocentric governance models provide a clear division of responsibilities and roles among institutions, allowing for more pragmatic decision-making than polycentric models. Well-defined hierarchies can provide the framework for efficient management through established methods of collaboration. Responsibility for outcomes is clear, and reduced complexity can speed up urgent decisions (Termeer et al. 2010). Monocentric systems may involve public participation, but in a more straightforward manner, with a single center of power providing feedback (Morrison et al. 2019). Access to public funding may also be facilitated through this model. Despite these advantages, monocentric systems often have limited institutional cooperation and inadequate citizen participation. Certain actors may be excluded or consulted late in projects with limited opportunities to intervene. These monocentric models are often governed by rules set by government institutions and not adaptable to local conditions (Edelenbos et al. 2021). The dominance of a single decision maker may also oversimplify problems (Van Zeijl‐Rozema et al. 2008), thereby neglecting important interactions that are necessary when it comes to restoration. This can lead to unintended consequences, such as the drying up of wetlands due to hydraulic projects or conflicts with farmers due to efforts to rewet wetlands. Monocentric governance can ignore local needs and cause an uneven distribution of restoration benefits, often involving stakeholders late in the governance processes, potentially causing increased skepticism and rejection by local actors.

Community-based models are driven by highly motivated and charismatic actors (often volunteers) committed to addressing persistent local problems (Edelenbos et al. 2021), demonstrating strong attachment to values and local areas, and proactive involvement in decision-making. Community based models can influence and support local governments through active partnerships (Henfrey et al. 2023). The diversity of membership enhances their performance by providing redundancy, capacity diversification and often gives higher importance to intangible values associated with nature. This can help maintain stability even as leaders change. They support networks of volunteers, ecologists, consultants, and scientists, strengthening community ties and collaborative efforts (Flood et al. 2022). The community-based scale inherently makes this type of governance system less suitable for large wetlands, especially those that cross administrative or national boundaries. Additionally, this system requires considerable effort and relies heavily on individual skills and intermediaries. Success is often dependent on the community’s ability to obtain and use resources and manage complex grant applications (Flood et al. 2022). To address these difficulties, community-based systems often partner with national organizations, NGOs, or other networks for collaboration, resource sharing, and mutual support (Henfrey et al. 2023). Community-based restoration may struggle with inconsistent resources, including financial support and fluctuating availability of volunteers (Flood et al. 2022). Leadership is also critical (Edelenbos et al. 2021): reliance on strong leaders can make the system unstable over time.

In sum, governance networks can promote restoration efforts by facilitating interactions among organizations, agencies, and other stakeholders involved in decision-making and action (Alexander et al. 2016). This model connects people and places, facilitating knowledge exchange and mutual learning. Network models provide the possibility for individual stakeholders to gain visibility, creating further connections and collaborations, and enhancing the potential of new and improved projects. Networks do provide resources, but are also more demanding in terms of time and capacity. Developing international relationships is a long-term investment, and results may not be immediate. Governance systems that depend on networks and cooperation may not be effective for urgent issues. Like polycentric systems, networking governance systems require openness to collaboration, which can be an additional challenge. If collaboration mechanisms are not well established, the benefits of this governance system may be diminished or lost.

## Conclusion

This research has shown that there is not a single governance model that can be directly replicated in every restoration site and most of the sites use a fuzz of one or more models. Each model has its advantages and disadvantages, and must be adapted to the specific context. The adaptation and combination of models is recommended to promote appropriate governance that will allow for effective ecological restoration and provide opportunities for upscaling in the future. Independent of the governance structure in place, it is essential to understand and manage stakeholder expectations and understand the power balances. All governance models rely on the co-creation of restoration measures, by bringing together heterogeneous actors, or addressing one of the major challenges of restoration: closing the gap between those who are potential beneficiaries of the ecosystem services provided by wetlands and that value them and those that have the authority to manage ecological restoration.

Through this research, the WaterLANDS project has formulated recommendations featured in the Theoretical Governance Framework for successful ecological restoration of wetlands that can help wetland practitioners, local community members and authorities remove some of their barriers to wetland restoration and to compensate for the weaknesses of the various existing governance models (https://planbleu.org/en/publications/waterlands-characterising-supportive-governance-and-policy/).

## Supplementary information

Supplementary information1

Supplementary information2

## Data Availability

No datasets were generated or analysed during the current study.
