# Supplementary material for: Characterizing governance models for upscaling wetland restoration
Source: Environ Manage. 2025 Mar 3;75(5):1155–67. doi: 10.1007/s00267-025-02132-2 (PMC12033105; doi:10.1007/s00267-025-02132-2)
Supplement: Supplementary file 1 — Supplementary information1 [file 267_2025_2132_MOESM1_ESM.docx]

The specific survey questions were:

1. Geographical location?
2. When did the first restoration effort take place?
3. Total surface of the restoration effort (km²)?
4. What are the main ecosystems concerned (e.g. peatlands, bogs, salt marshes, fens...)? Use keywords.
5. Before the restoration works, had the main factors that were degrading the zone been identified? Were they natural or human-induced?
6. What type of restoration works undertaken?
7. Stakeholders and key economic sectors (type and level)?
8. Land tenure model (private/public/blended)?
9. What are the main sources of funding that you applied to or obtained for the restoration activities?
10. Please indicate project names, implementation timelines...
11. Has a restoration project committee involving all categories of local stakeholders been created?
12. Are restoration results related to governance available (project reports, scientific articles, web page, links, etc..)?
13. Has a monitoring system been established? By whom (e.g. researchers, citizen science...)?
14. Do local communities and actors consider that the ecosystem restoration effort has been/is currently successful?
15. Do you think that this restoration effort increased the resilience: of your territory/your communities to the anthropogenic impacts of environmental degradation and climate challenge?
