# Supplementary figures and images for: Characterizing governance models for upscaling wetland restoration
Source: Environ Manage. 2025 Mar 3;75(5):1155–67. doi: 10.1007/s00267-025-02132-2 (PMC12033105; doi:10.1007/s00267-025-02132-2)

1. **Monocentric model:**


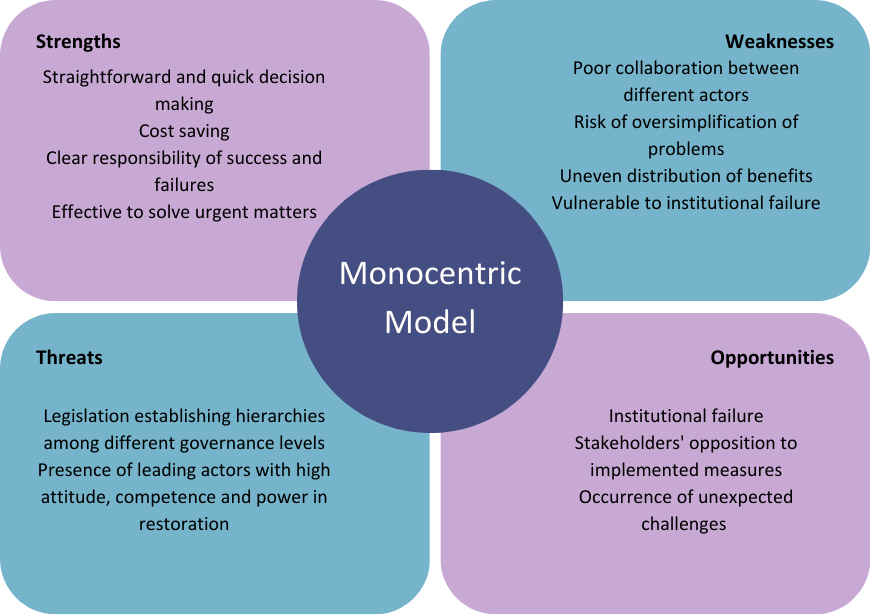


1. **Polycentric model:**


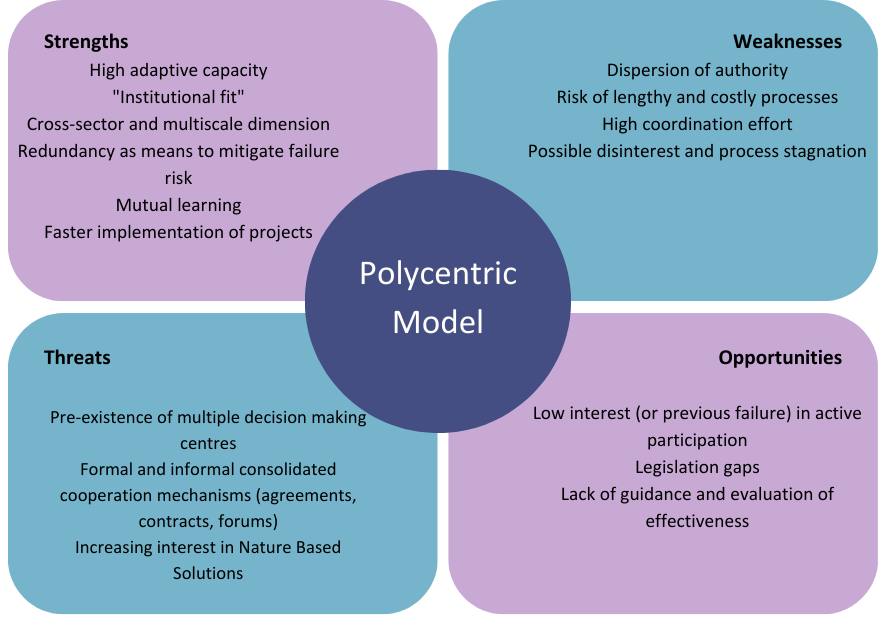


1. **Community-based model:**


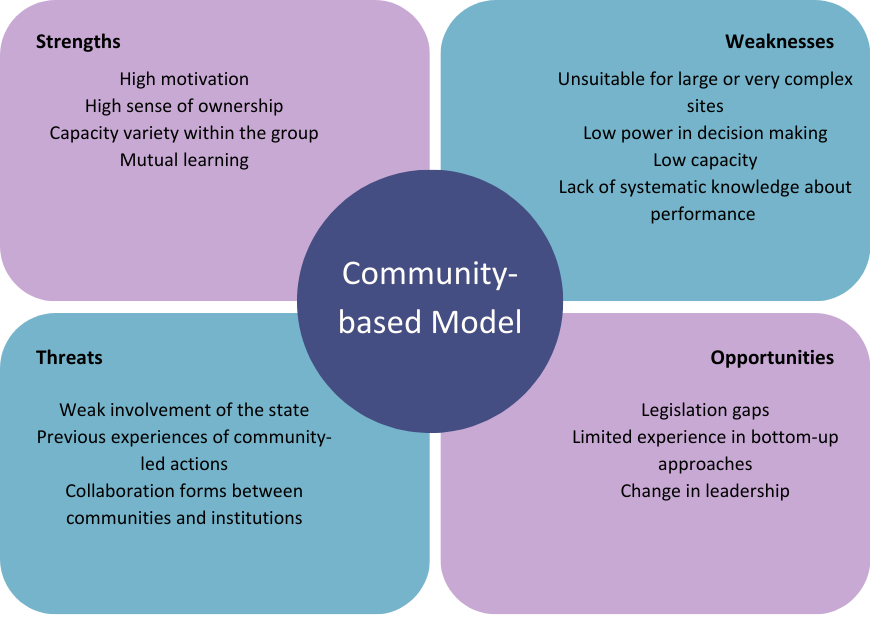


1. **Networking model:**


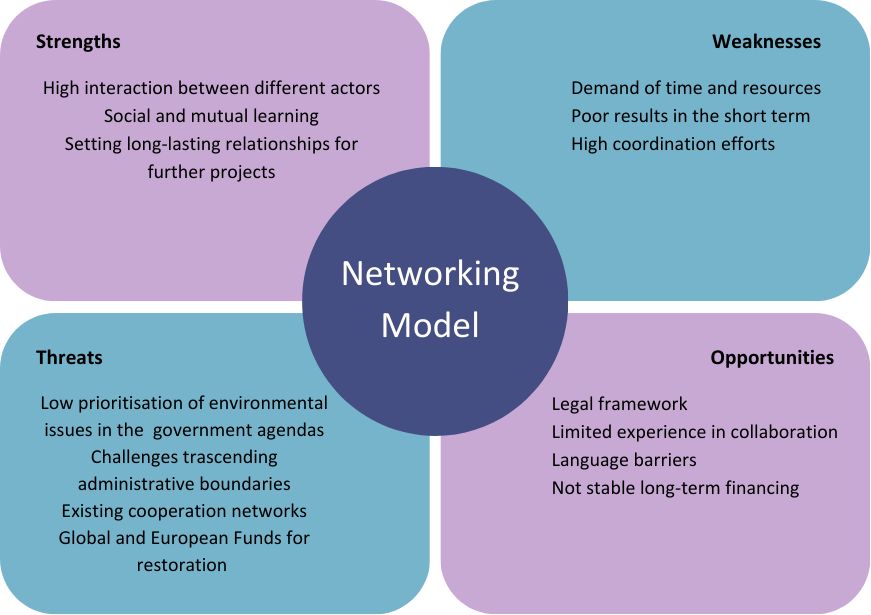

Supplement: Supplementary file 2 — Supplementary information2 [file 267_2025_2132_MOESM2_ESM.docx]
